# Temporal Trends in Metal Pollution: Using Bird Excrement as Indicator

**DOI:** 10.1371/journal.pone.0117071

**Published:** 2015-02-13

**Authors:** Åsa M. M. Berglund, Miia J. Rainio, Tapio Eeva

**Affiliations:** Section of Ecology, University of Turku, Turku, Finland; University of Vigo, SPAIN

## Abstract

Past mining and smelting activities have resulted in metal polluted environments all over the world, but long-term monitoring data is often scarce, especially in higher trophic levels. In this study we used bird (*Parus major* and *Ficedula hypoleuca*) excrement to monitor metal pollution in the terrestrial environment following 16 years of continuously reduced emissions from a copper/nickel smelter in Finland. In the early 1990s, lead and cadmium concentrations dropped significantly in excrement, but the reduction did not directly reflect the changes in atmospheric emission from the smelter. This is likely due to a continuous contribution of metals also from the soil pool. We conclude that bird excrement can be used to assess changes in the environment as a whole but not specifically changes in atmospheric emission. Inter-annual variation in excrement concentration of especially copper and nickel demonstrates the importance of long-term monitoring to discern significant trends.

## Introduction

Mining and smelting operations have resulted in increased metal concentration in the environment [1], with negative effects on the surrounding ecosystem as a result [2–6]. However, a combination of environmental law enforcements, regulations and technological developments have resulted in continuously reduced atmospheric emissions from such industries during the last decades [7]. Although smelter sites have been vastly studied, information from long-term monitoring in these environments is scarce, especially regarding species of higher trophic level.

In terrestrial systems, metals can be stored in the upper soil pool [8,9], thus contaminating the environment over a prolonged time period. Long-term monitoring of contaminated environments have indicated that despite emission reductions, vegetation continue to accumulate high concentrations of metals close to point sources, as a combination of uptake from polluted soils via roots, as well as surface accumulation through soil dust [10]. Either way, there is a risk for continued contamination for leaf-eating herbivores and transport via the terrestrial food-chain.

Birds are useful bioindicators for exposure to pollutants such as metals [11], and there is an emerging number of studies using small passerines in ecotoxicological studies around metal industries (see for example [4,12–15]). In order to get further knowledge on the long-term changes in polluted environments we used excrement from nestling pied flycatcher (*Ficedula hypoleuca*) and great tit (*Parus major*) to study element (cadmium [Cd], copper [Cu], nickel [Ni], and lead [Pb]) exposure during 16 years of significant emission reductions from a Cu/Ni smelter in one of the most polluted areas in Finland [16]. Bird excrements have been suggested as useful non-destructive indicators of metal contamination in birds’ diet and environment [17–19] and excrements are conveniently easy to collect from nest box breeding species such as pied flycatcher and great tit. The aim was i) to monitor long-term changes in metal exposure and ii) to determine the use of bird excrement as a proxy for atmospheric metal emission.

## Materials and Methods

### Study area and data collection

**Fig 1:**
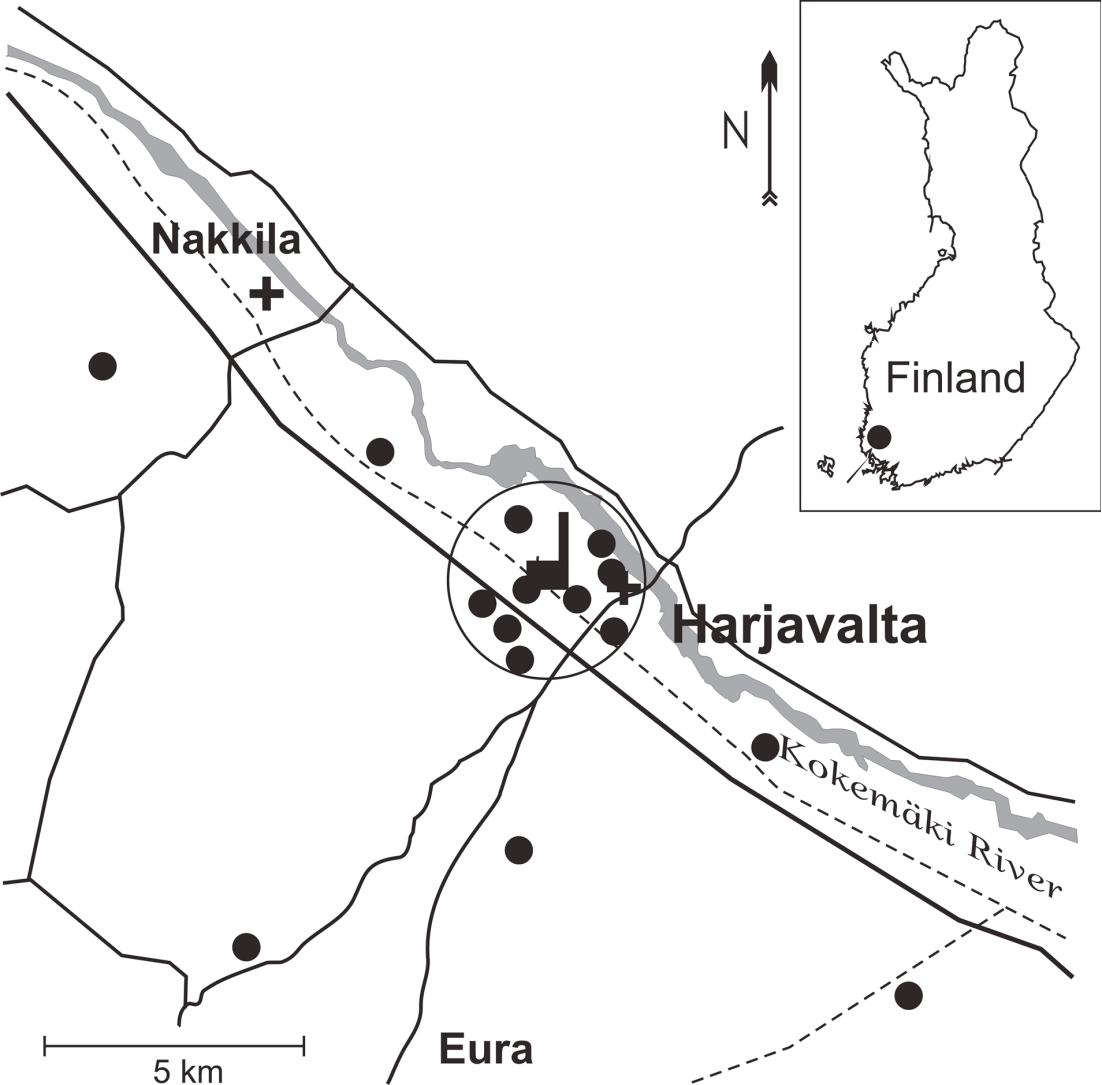
Map of the study area and position of the smelter and the fifteen sites (black dots) where nestling birds were sampled. Circle indicates zone 1, sites within 2 km from the smelter, and plus signs indicate city locations.

**Fig 2:**
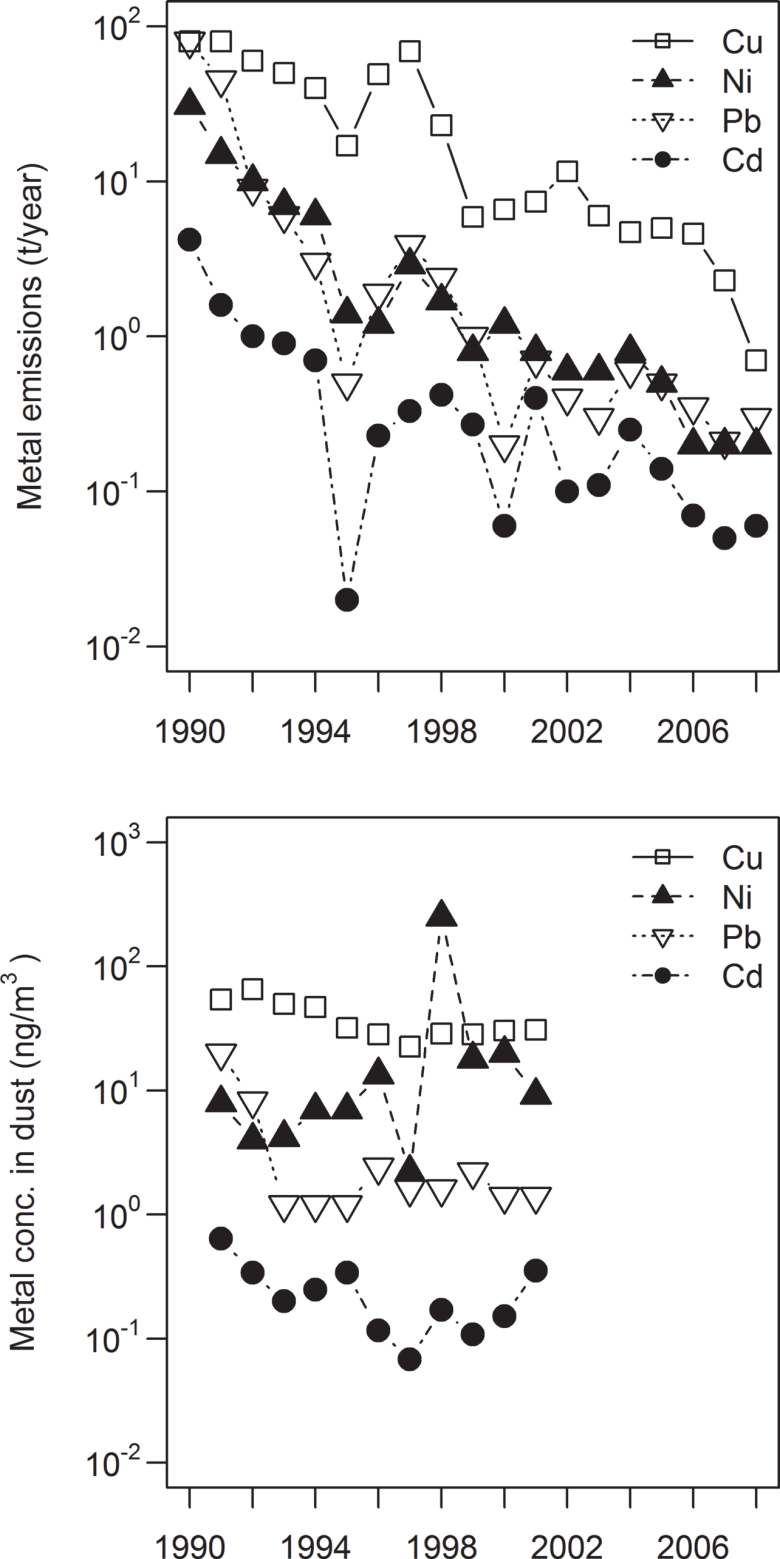
Yearly cadmium (Cd), copper (Cu), nickel (Ni) and lead (Pb) emissions (t year^-1^) from the Cu/Ni-smelter in Harjavalta (a) and average summer (April–August) concentration in aerial dust, collected in the town of Harjavalta (b). Emission data from Kozlov [7] and dust data from Harjavalta town.

This study was conducted between 1992 and 2008 (with some missing years) in the surroundings of the Cu/Ni smelter in Harjavalta (61˚20′N, 22˚10′E), in SW of Finland (Fig. 1). The smelter has been in operation since 1945 and sulfuric oxides and metals (especially arsenic [As], Cd, Cu, Ni, Pb and zinc [Zn]) are common pollutants in the vicinity of the smelter [20]. In 1992 1t Cd, 60t Cu, 10t Ni, and 9t Pb were emitted yearly to the atmosphere but from 1992 to 2008 metal emissions from the smelter have been reduced by 94–99% (Fig. 2a), due to installations of new filters in 1990, 1991 and 1994 [21] and building of a new, taller (140 m) stack in 1994 [22]. Apart from improved treatment of dust from chimneys, uncovered slag heaps occurred in the early 1990’s, contributing to wind-born metal rich dust. Thus, the most marked reduction in emission took place in the early 1990s (Fig 2a). Moss surveys also confirm this emission decline around Harjavalta, as a combination of decreased long-range transport and local emission from the smelter [23]. In 2008 slag heaps were still present as noise barriers, but vegetated by grass and trees, and slag was in sludge form in land basins. All of these improvements generally decreased the metal content of dust measured in Harjavalta town (Fig 2b). Bird populations have been extensively studied in the area since 1991. Among the observed effects are reduced breeding performance, growth abnormalities, reduced food abundance and reduced species diversity [24–27].

When handling nestlings for banding, excrement samples were collected fresh from defecating pied flycatcher (age of 6–13 days, mean: 7.5 days) and great tit nestlings (age of 5–15 days, mean: 7.7 days) into plastic tubes and stored in -20°C until they were dried for analyses. Nestlings were sampled at 15 study sites, each with 40–50 nest boxes at a distance of 0.6–11.2 km from the smelter, in three main directions (SW, SE and NW) (Fig. 1). Handling of birds were approved by the Ministry of Environment (5976/434/87, 24/4342/92, 11/57/22/97, 17/57/13/2002; http://www.ym.fi), Ministry of Agriculture and Forestry (3127/772/MMM1987, 2653/771/MMM1992, 2901/77/97, 2324/722/2002; http://www.mmm.fi) and Environment Center of South-West Finland (LOS-2007-L-1001–254; http://www.syke.fi). No ethical approval is needed for this non-invasive sample collection as the distress is less than the pain caused by the induction of a needle (Ministry of Agriculture and Forestry, Finland; http://www.mmm.fi). Samples from nestlings of the same brood were combined. In total, 178 pied flycatcher broods and 325 great tit broods were sampled. The study sites had similar forest habitats and the forests in the area are dominated by Scots pine (*Pinus sylvestris*), which forms mixed stands with spruce (*Picea abies*) and birches (*Betula* spp.) (for more information see [26]). Due to current and long-term deposition, elevated heavy metal concentrations in soils occur in the vicinity of the smelter with exponentially decreasing metal concentrations with increasing distance from the industry [20], reaching background levels between 4 and 8 km from the smelter [28,29]. Nest box areas were thus divided into two pollution zones, zone 1 within the close vicinity of the smelter (<2 km from the smelter) and zone 2 (>4 km), the reference zone, with less impact from the smelter. Both pied flycatchers and great tits have small territories and forage within 100 and 300m, respectively, of their nests [30,31].

### Metal analyses

Fecal sacs were dried at 50˚C for 72 hours. Two milliliters of supra-pure HNO_3_ (supplied by Merck) were added to the samples in Teflon bombs for digestion with a microwave system. After digestion, samples were diluted to 50 ml with de-ionized water. Several studies have been performed in the study area, thus fecal samples have been collected and analyzed yearly, with the available technology (AAS and ICP-MS). Several studies have suggested that the two methods are comparable [32–34], although the detections limits differ (lower for ICP-MS) [35,36]. Element concentrations (Cd, Cu, Ni, and Pb) were determined by flame AAS (PerkinElmer 603 in 1992 and 3100 in 1993 and 1994) in 1992–1994, graphite furnace AAS (PerkinElmer 3100) in 1999 (and for Cd in 1993) and ICP-MS (Elan 6100 DCR+ from PerkinElmer-Sciex) in 2000–2008, although not all elements are represented each year. At a maximum, metal analysis were available from three years in the 1990s and four years in the 2000s. Samples below detection limit in 1992–1994 (using GF-AAS) were assigned a new value calculated with log-probit regression [37,38]. In 1992, values below detection limit were assigned 3.06 and 1.74 μg g^-1^ for Cd and Pb respectively. In 1993, values below detection limit were assigned 0.35, 1.24, and 1.24 μg g^-1^ for Cd, Ni, and Pb respectively, and in 1994 they were assigned 4.13 μg g^-1^ for Pb. We calculated unique values for each year, to minimize errors from using different PerkinElmer models and possible differences in sensitivity of the machine. Individual excrement concentrations are available as Supporting information (S1 Table).

The calibrations of the instruments were done with certified solutions (Titrisol for Cd, Cu, Ni, and Pb in 1992–1999; Claritas PPt, Multi element solution 2A from Spex Certiprep in 2000–2008). Samples from 2005 and 2008 were analyzed in batches with certified reference material (mussel tissue ERM-CE278), with recovery rates within 10% of the certified value (Table 1). Certified reference material was not included in the analyses from the 1990s, but samples from 1993, 1999 and 2005 were analyzed with an internal standard (excrement from wintering great tits; Table 1). Thus the internal standard has been analyzed with both AAS and ICP-MS, and can, to some extent, be used to validate the two methods.

**Table 1:** Element concentrations^a^ (mean ± SE) of reference samples of wintering adult great tits (excrement) used as an internal standard, and certified reference material ERM-CE278 (mussel), run in batches with samples from different years.

	Excrement			Mussel		
	1993	1999	2005	2005	2008	certified value
Cd	2.25 ± 0.05 (5)	NA	2.24 ± 0.01 (5)	0.34 ± 0.01 (4)	0.35 ± 0.01 (5)	0.35 ± 0.01
Cu	342 ± 12.0 (5)	421 ± 6.49 (6)	471 ± 6.81 (5)	9.62 ± 0.03 (4)	9.95 ± 0.08 (5)	9.45 ± 0.13
Ni	60 ± 2.74 (5)	NA	53 ± 2.56 (5)	0.57 ± 0.01 (4)	1.17 ± 0.04 (5)	NI
Pb	26.5 ± 1.46 (5)	22.2 ± 1.40 (6)	20.2 ± 1.28 (5)	2.10 ± 0.01 (4)	2.05 ± 0.02 (5)	2.00 ± 0.04

The certified values for mussel tissue (cert value) obtained from the Institute for Reference Materials. Sample sizes are given in brackets.

^a^ μg g^-1^; dw

^NA^ Not analyzed

^NI^ Element not included in certified reference material

To enable comparisons of excrement metal concentrations in this study (on a dry weight basis) with literature showing only fresh weights, a conversion factor of 4.75 ± 0.36 (n = 5) was used for pied flycatcher excrement and 4.83 ± 0.16 (n = 69) was applied for great tits. These values were obtained from excrement samples collected in 2008 and 2011, based on their weights prior to, and after drying.

### Statistical analyses

Statistical analyses were performed with SAS statistical software 9.2 [39]. Differences in excrement metal concentrations were analyzed for each species separately, using general linear models (GLIMMIX procedure in SAS) with normal error distribution and identity link function, followed by post hoc comparisons (least squares means) using Tukey-Kramer adjustment for multiple comparisons. Concentrations were log-transformed (to fit normal distribution) and models included year and pollution zone (zone 1 vs. 2) as categorical fixed factors, and the interaction term year × zone. If interaction was not significant, the model was simplified (excluding the interaction term), given that the simplified model had a lower AIC-value. The concentrations of Cu were unreasonably high in two excrement samples from 1993 (11900 and 9312 μg g^-1^ for a pied flycatcher and great tit respectively) and the concentration of Pb in one great tit in 2005 (580 μg g^-1^). Those were considered outliers and omitted from all analyses. The level of statistical significance was set to p < 0.05.

Correlations between metal emissions, dust and excrement samples were calculated using Kendall rank correlation. Yearly emission data between 1991 and 2008 (partly published in [7]), and data on metal content in dust collectors (i.e. the air quality) between 1991 and 2001 were obtained from Harjavalta town. The latter was calculated as a summer average (April–August), to reflect the breeding period. For excrements, the average for polluted sites was used for each year. Only models including at least 4 years were included, thus no correlation test was performed between dust and excrement (n = 1–3).

## Results

### Pied flycatcher

**Fig 3:**
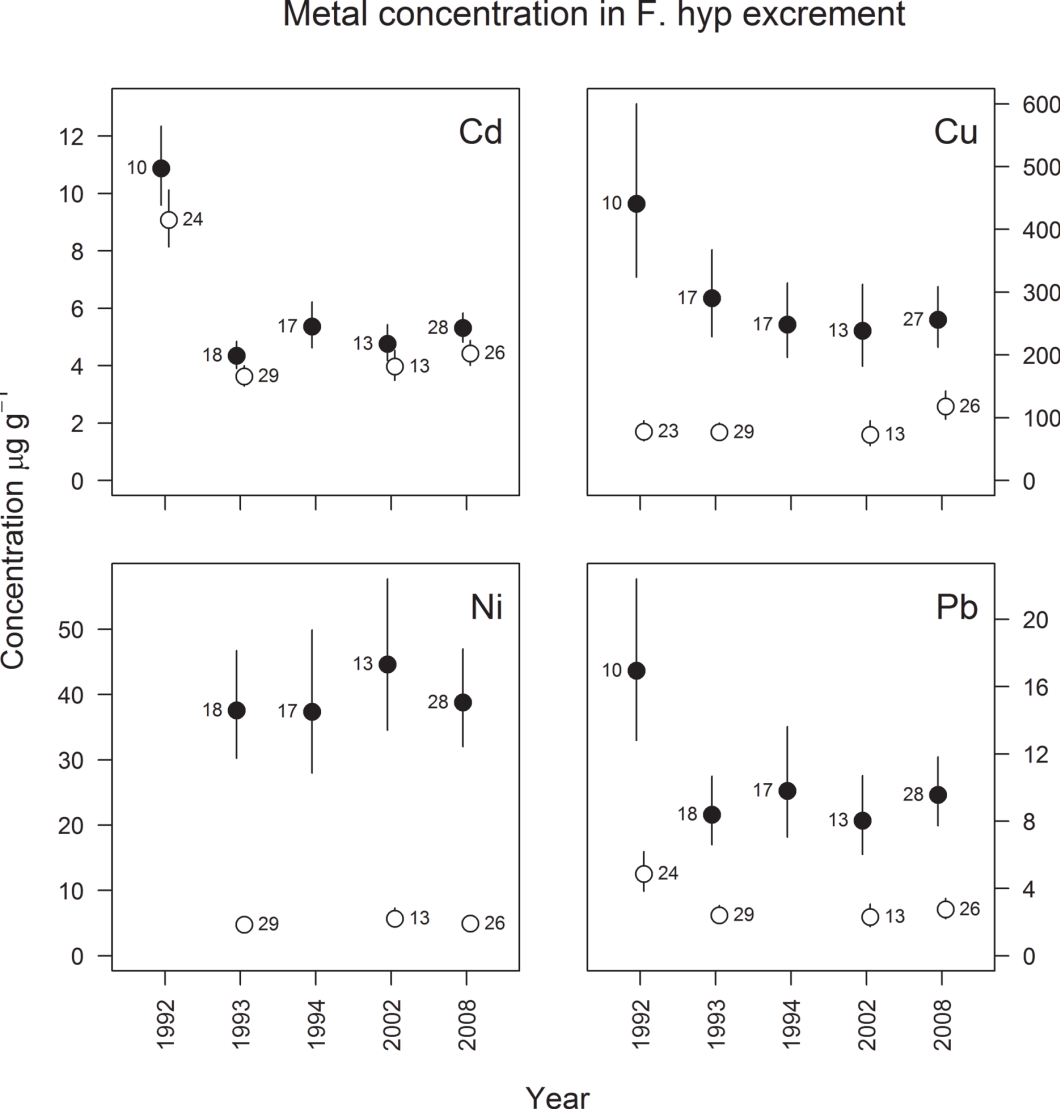
Metal concentration (μg g^-1^ dw, geometric mean ± 95% confidence limit) in excrement of pied flycatcher (*Ficedula hypoleuca*) nestlings sampled between 1992 and 2008 from polluted (black dots, ≤ 2 km from the smelter) and reference areas (open dots, > 4 km from the smelter). Numbers denote sample size.

There were spatial differences in fecal element concentration of pied flycatcher nestlings (Cd: F_1,172_ = 13.0, p<0.001; Cu: F_1,166_ = 222, p<0.0001; Ni: F_1,139_ = 364, p<0.0001 and Pb: F_1,172_ = 125, p<0.0001), with higher concentrations in the vicinity (≤ 2 km) than farther (>4 km) from the industry (Fig. 3). The difference between Cu concentrations in pied flycatcher excrement from zone 1 and 2 was greater in 1992 than the rest of the years (significant interaction, F_3,166_ = 6.2, p<0.001).

There were also annual differences in Cd, Pb and Cu concentration of pied flycatchers excrement (F_4,172_ = 48, p<0.0001; F_4,172_ = 6.4, p<0.0001 and F_4,166_ = 2.7, p<0.05, respectively). The concentration of Cd was significantly higher in 1992 (11 and 9.1 μg g^-1^ Cd in zone 1 and 2, respectively) than the following years (p_adj_<0.0001 for all), and the concentrations were also significantly lower in 1993 than in 2008 (p_adj_<0.02) (Fig. 3). As an example, the mean Cd concentrations decreased by 60% between 1992 and 1993 in both zone 1 and 2 (4.4 and 3.6 μg g^-1^ Cd respectively in 1993), and remained constant throughout the 2000s. The concentrations of Pb were significantly higher in 1992 (17 and 4.9 μg g^-1^ Pb in zone 1 and 2 respectively) than in all other years but 1994 (p_adj_<0.003 for all but 1994). The concentration of Pb decreased by 50% between 1992 and 1993 in zone 1 and 2 (8.4 and 2.4 μg g^-1^ Pb in 1993), and remained at similar levels throughout the 2000s. In zone 2, Cu concentrations were significantly lower (p_adj_<0.002) in 1993 (77 μg g^-1^) than in 2008 (118 μg g^-1^). Nickel concentrations in pied flycatcher excrement did not differ significantly between years (Fig. 3).

### Great tit

**Fig 4:**
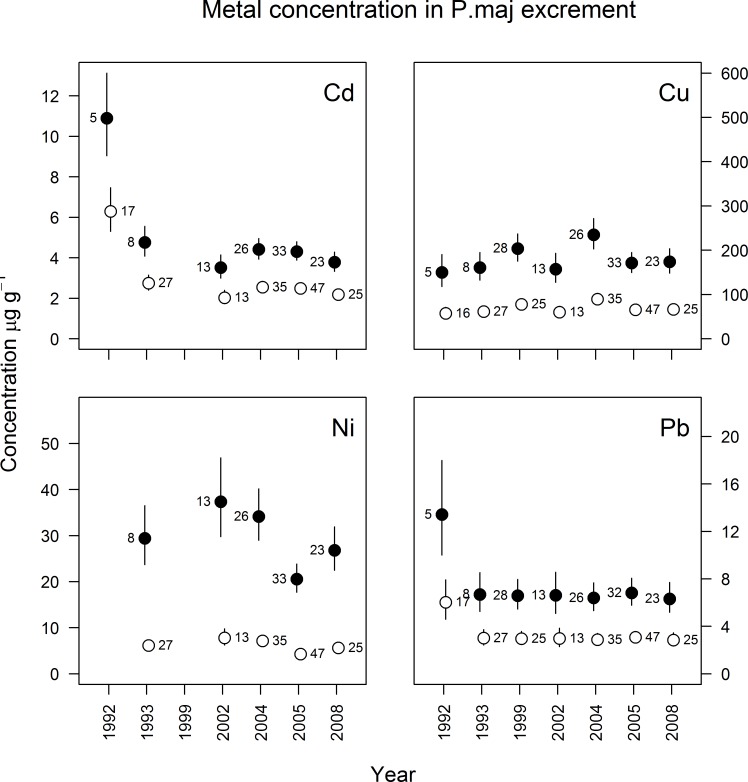
Metal concentration (μg g^-1^ dw, geometric mean ± 95% confidence limit) in excrement of great tit (*Parus major*) nestlings sampled between 1992 and 2008 from polluted (black dots, ≤ 2 km from the smelter) and reference areas (open dots, > 4 km from the smelter). Numbers denote sample size.

Spatial differences were also found in great tit nestling excrement and birds from the vicinity of the smelter (<2 km) had significantly higher concentrations of Cd (F_1,265_ = 116, p<0.0001), Cu (F_1,316_ = 262, p<0.0001), Ni (F_1,244_ = 461, p<0.0001) and Pb (F_1,317_ = 119, p<0.0001) than nestlings farther from the smelter (Fig. 4).

Annual differences occurred for all analyzed metals in great tit excrement (Cd: F_5,265_ = 25, p<0.0001; Cu: F_6,316_ = 4.3, p<0.001; Ni: F_4,244_ = 9.8, p<0.0001; Pb: F_6,317_ = 3.8, p<0.002). The excrement concentration of Cd and Pb were highest (Cd: p_adj_<0.0001 for all, and Pb: p_adj_<0.004 for all) in 1992 (mean of 11 and 13 μg g^-1^, respectively in zone 1). By 1993 the concentrations decreased by 56% and 50% and remained at similar levels throughout the 1990s and 2000s. The essential trace element Cu varied little between years, but in 2004 the excrement concentrations were significantly higher (235 and 90 μg g^-1^ Cu in zone 1 and 2, respectively) than in 1992, 1993, 2002 and 2005 (p_adj_<0.05 for all). Nickel concentrations were significantly lower in 2005 (21 and 4.3 μg g^-1^ for zone 1 and 2, respectively), compared to all analyzed years but 1993 (p_adj_<0.02).

### Correlation with atmospheric emissions

Metal concentration (geometric mean) of pied flycatcher excrement did not correlate with yearly emissions from the smelter, but the concentration of Cd in great tit excrement and emissions did (Table 2). Due to limited data on metal concentration in dust, no correlation test was preformed between excrement and dust. Metal concentrations in dust collectors did not correlate with metal emissions from the smelter (Table 2). For example, the peak of Cu emissions in 1997 was not observed in dust collectors at Harjavalta town and the peak of Ni in dust in 1998 was not observed in the emission data (Fig. 2).

**Table 2:** Kendall tau rank correlation coefficients (τ), p-value and sample size (N) for correlations between metal content (cadmium [Cd], copper [Cu], nickel [Ni] and lead [Pb]) of atmospheric emission (t y^-1^) and concentrations in pied flycatcher (*F*. *hyp*) and great tit (*P*. *maj*) excrements (μg g^-1^; dw) and dust collectors (ng m^-3^).

	Emissions											
	Cd			Cu			Ni			Pb		
	τ	p	N	τ	p	N	τ	p	N	τ	p	N
*F*.*hyp*	0.20	0.82	5	0.60	0.23	5	-0.33	0.75	4	0.40	0.48	5
*P*.*maj*	**0.87**	**0.02**	**6**	-0.62	0.07	7	0.20	0.82	5	0.53	0.14	7
*Dust*	0.18	0.47	11	0.22	0.37	11	-0.46	0.07	11	0.19	0.46	11

Significant correlations are shown in bold.

## Discussion

The metal content in bird excrement reflects the metal concentration in food and the ambient environment [17–19,40]. Thus, our results show that birds close to the smelter are exposed to high Cd, Cu, Ni and Pb concentrations. This is also reflected in reduced breeding performance and local survival of adult birds in the vicinity of the smelter [25,41]. The environmental exposure of Cd and Pb dropped markedly between 1992 and 1993 and remained relatively constant after 1993. Although metal content in excrement and internal tissues do not necessarily correlate [42], the same decrease was found in liver and bone of nestlings from the same area [14,43]. Similar to our results, Eeva and Lehikoinen [14] showed that the most pronounced reduction in lead bone concentration took place between 1991 and 1994 (birds were not analyzed in 1992 nor 1993). This indicates that there was a significant reduction in metal exposure (and accumulation) in the early 1990s when emissions were highest during this study (Fig 2a). Although atmospheric emissions from the smelter have decreased over the past decades, there were weak correlations between yearly emissions and metal concentration in excrement (except for Cd in great tit). Thus, the environmental exposure to birds was not directly related with the atmospheric emission, a result which is in concordance with other studies [4,44].

The severity of soil pollution has been suggested as one key to recovery of populations after atmospheric emissions decline [3,43–45]. As a consequence, atmospheric emissions may be a greater source of exposure when emissions are high (seen in 1992), but their relative importance decreases as emissions decline. Thus, in areas with a history of metal pollution, the soil pool will have a greater impact at lower atmospheric emissions of metals. The metal content of soils in the study area were comparable in 1992 and 2005 [46], indicating high and stable concentrations of metals in soils throughout the study period. This may be one explanation for the stable concentrations in bird excrement since 1993. This would also explain the lack of strong correlation between excrement and emissions. Hence, we propose that bird excrement most likely reflect the current and past metal emissions, as a combination of atmospheric deposition and metals stored in the soil. Thus, excrement from terrestrial birds are suitable for long-term monitoring of environmental changes of metal concentrations, but not atmospheric metal emission *per se*. Also, our results show the importance of monitoring higher trophic levels, as there is a risk for continued accumulation within the food-chain.

Metal concentrations in excrement reflect the unabsorbed metal content in food items and excretion of absorbed metals [18] and food items have been suggested as an important source of metals to insectivorous birds [44,47]. Both pied flycatchers and great tits are insectivorous during their nestling phase, though the great tit is a caterpillar-specialist whereas the pied flycatcher is more of a generalist [26,48]. Berglund et al. [44] suggested that in highly polluted environments, the contribution of metals from soils may be of greater importance than the atmospheric input. Thus, changes in atmospheric emissions will not be directly related with the metal concentration in the environment, but rather a mixture of metals with atmospheric and soil origin. Soil metal pool might not only be a major source of metal contamination on vegetation, but it might also pose a risk for continued contamination to the terrestrial food chain [10].

The concentration of Pb and Cd was high also in the reference sites (zone 2) in 1992 compared to the following years. This resembles the general trend in Finland and Europe with declining deposition and most pronounced decreases in the late 1980s—early 1990s [23,49]. Although smelter emissions reach the background areas to some extent, it is unlikely that emission from the smelter is the major source of metals in these areas. In the 2000s the excrement concentrations of Cd and Pb were comparable with excrement from unpolluted sites in Europe [4,12,13,44], indicating minor contribution from the smelter in the 2000s. Thus, reduced concentrations are expected also in excrement from the reference site in the early 1990s, as a consequence of a general decline in metal deposition. Reduced background concentrations of Pb in the 1990s is most likely a result of phasing out leaded petrol. In Finland it has not been used since 1994.

The inter-annual variation of especially Ni and Cu in excrement show the importance of including more than one year of sampling to predict environmental contamination, as has been stressed by others [10,50]. Environmental factors, such as temperature and precipitation, might also affect metal accumulation [10], contributing to the annual variation. This might be of special importance for mobile elements such as Ni [28], which might be more bioavailable due to increased solubility, but also less bioavailable if it is washed down into deeper soil layers. The latter might be more probable in our study system, as fecal Ni concentrations in the vicinity of the smelter are among the highest reported in passerines, even in the 2000s [4,12,13,17]. Ni also accumulated in liver tissue at concentrations considered toxic [43].

Annual differences in diversity and density of food items may contribute to the yearly variation in metal exposure, as invertebrate species (food items for birds) differ in metal concentration [51–53]. For example, spiders are known to contain high levels of Cu because their haemocyanin is rich in Cu [51,53] and the proportion of spiders in pied flycatcher diet has been shown to differ between years [51]. Therefore some of the yearly variation in Cu levels could be due to variable proportion of spiders in nestling diet. Also, even though Cu is an essential element, hence regulated in birds and their invertebrate prey, different species have different requirements, which result in varying Cu concentrations in nestling excrement depending on the ingested invertebrate species. An additional source of inter-annual or sample variation is grit ingestion, or grit adhered to ingested food items that would result in higher metal content in samples containing grit. As a consequence of all the above, relatively large sample sizes are needed to determine and predict significant changes. Depending on the specific diet of bird species, they are exposed to varying concentration of metals merely as a consequence of the accumulation in their food items.

To conclude, this study shows that bird excrement can be a useful tool for long-term monitoring in polluted areas, as it reflects the combined exposure of past and present deposition from point-sources, i.e. the total environmental exposure at higher trophic levels. It can however not be used as a proxy for atmospheric metal emissions *per se*. The annual and individual variation of metal concentrations highlights the importance of relatively large sample sizes when using excrement samples.

## Supporting Information

S1 Table Individual metal concentration in bird excrement. (DOCX)
