# Supplementary material for: Temporal Trends in Metal Pollution: Using Bird Excrement as Indicator
Source: PLoS One. 2015 Feb 13;10(2):e0117071. doi: 10.1371/journal.pone.0117071 (PMC4332472; doi:10.1371/journal.pone.0117071)
Supplement: S1 Table — (DOCX) [file pone.0117071.s001.docx]

**Supporting Information**

**Table S1.** The individual metal concentration in bird excrement from different years and distances to the smelter. Each row represent samples from one brood. The columns of the dataset are as follows: year of sampling, distance to the smelter, analytical method, type of sample (feces or reference), species (*Ficedula hypoleuca* or *Parus major*), concentration of Cu, Ni, Pb and Cd (all shown in ppm (dw)). Samples under detection limit are denoted UDL.

| **Year** | **Dist** | **Method** | **Sample** | **Spec** | **Cuppm** | **Nippm** | **Pbppm** | **Cdppm** |
| --- | --- | --- | --- | --- | --- | --- | --- | --- |
| **1992** | 0.92 | AAS | FECES | FICHYP | 498.00 | . | 48.00 | 8.20 |
| **1992** | 0.92 | AAS | FECES | FICHYP | 355.00 | . | UDL | 15.00 |
| **1992** | 0.92 | AAS | FECES | FICHYP | 891.00 | . | 47.40 | 11.90 |
| **1992** | 0.81 | AAS | FECES | FICHYP | 501.00 | . | 69.70 | 6.50 |
| **1992** | 0.81 | AAS | FECES | FICHYP | 570.00 | . | UDL | 14.60 |
| **1992** | 0.81 | AAS | FECES | FICHYP | 399.00 | . | 20.50 | 11.30 |
| **1992** | 0.96 | AAS | FECES | FICHYP | 495.00 | . | 10.30 | 13.40 |
| **1992** | 1.20 | AAS | FECES | FICHYP | 261.00 | . | 19.60 | 7.30 |
| **1992** | 1.20 | AAS | FECES | FICHYP | 295.00 | . | 22.30 | 6.80 |
| **1992** | 1.20 | AAS | FECES | FICHYP | 397.00 | . | 22.70 | 7.50 |
| **1992** | 5.10 | AAS | FECES | FICHYP | 132.00 | . | UDL | 10.20 |
| **1992** | 5.10 | AAS | FECES | FICHYP | 87.00 | . | UDL | 5.30 |
| **1992** | 5.10 | AAS | FECES | FICHYP | 135.00 | . | 6.80 | 7.00 |
| **1992** | 5.10 | AAS | FECES | FICHYP | 185.00 | . | 10.90 | 9.50 |
| **1992** | 5.10 | AAS | FECES | FICHYP | 123.00 | . | 7.70 | 9.70 |
| **1992** | 10.78 | AAS | FECES | FICHYP | 78.30 | . | 15.60 | 5.90 |
| **1992** | 10.78 | AAS | FECES | FICHYP | . | . | 9.60 | 8.00 |
| **1992** | 10.78 | AAS | FECES | FICHYP | 171.00 | . | 14.00 | 5.50 |
| **1992** | 10.78 | AAS | FECES | FICHYP | 22.20 | . | UDL | 10.10 |
| **1992** | 10.78 | AAS | FECES | FICHYP | 79.00 | . | UDL | 8.20 |
| **1992** | 10.01 | AAS | FECES | FICHYP | 21.90 | . | UDL | 9.10 |
| **1992** | 10.01 | AAS | FECES | FICHYP | 6.20 | . | 10.50 | 5.40 |
| **1992** | 10.01 | AAS | FECES | FICHYP | 68.00 | . | UDL | 6.40 |
| **1992** | 10.01 | AAS | FECES | FICHYP | 65.90 | . | 10.00 | 9.80 |
| **1992** | 4.38 | AAS | FECES | FICHYP | 63.20 | . | UDL | 10.60 |
| **1992** | 4.38 | AAS | FECES | FICHYP | 108.00 | . | 6.60 | 9.80 |
| **1992** | 4.38 | AAS | FECES | FICHYP | 187.00 | . | UDL | 7.90 |
| **1992** | 4.38 | AAS | FECES | FICHYP | 175.00 | . | UDL | 8.20 |
| **1992** | 4.38 | AAS | FECES | FICHYP | 78.20 | . | UDL | 10.20 |
| **1992** | 10.82 | AAS | FECES | FICHYP | 66.60 | . | UDL | 12.60 |
| **1992** | 10.82 | AAS | FECES | FICHYP | 66.70 | . | 5.50 | 7.30 |
| **1992** | 10.82 | AAS | FECES | FICHYP | 58.20 | . | UDL | 10.00 |
| **1992** | 10.82 | AAS | FECES | FICHYP | 116.00 | . | UDL | 6.20 |
| **1992** | 10.82 | AAS | FECES | FICHYP | 74.60 | . | UDL | 6.60 |
| **1992** | 0.92 | AAS | FECES | PARMAJ | 137.00 | . | 59.00 | 10.80 |
| **1992** | 0.92 | AAS | FECES | PARMAJ | 107.00 | . | UDL | 12.50 |
| **1992** | 0.92 | AAS | FECES | PARMAJ | 139.00 | . | 40.00 | 8.60 |
| **1992** | 0.81 | AAS | FECES | PARMAJ | 381.00 | . | 7.00 | 9.20 |
| **1992** | 0.96 | AAS | FECES | PARMAJ | 283.00 | . | UDL | 10.80 |
| **1992** | 5.10 | AAS | FECES | PARMAJ | 133.00 | . | 15.20 | UDL |
| **1992** | 5.10 | AAS | FECES | PARMAJ | 89.00 | . | UDL | 3.30 |
| **1992** | 10.78 | AAS | FECES | PARMAJ | 84.80 | . | UDL | 7.10 |
| **1992** | 10.78 | AAS | FECES | PARMAJ | 73.90 | . | UDL | 3.40 |
| **1992** | 10.78 | AAS | FECES | PARMAJ | 31.20 | . | UDL | 5.80 |
| **1992** | 10.78 | AAS | FECES | PARMAJ | 34.90 | . | 43.70 | 6.90 |
| **1992** | 10.01 | AAS | FECES | PARMAJ | 68.40 | . | UDL | 5.10 |
| **1992** | 10.01 | AAS | FECES | PARMAJ | . | . | 8.80 | 4.10 |
| **1992** | 10.01 | AAS | FECES | PARMAJ | 48.90 | . | 16.30 | 6.20 |
| **1992** | 4.38 | AAS | FECES | PARMAJ | 58.80 | . | 11.80 | 5.90 |
| **1992** | 4.38 | AAS | FECES | PARMAJ | 99.90 | . | 11.40 | 3.50 |
| **1992** | 4.38 | AAS | FECES | PARMAJ | 73.70 | . | UDL | UDL |
| **1992** | 4.38 | AAS | FECES | PARMAJ | 60.80 | . | UDL | 6.70 |
| **1992** | 10.82 | AAS | FECES | PARMAJ | 64.90 | . | 8.10 | 7.30 |
| **1992** | 10.82 | AAS | FECES | PARMAJ | 12.30 | . | 8.60 | 6.10 |
| **1992** | 10.82 | AAS | FECES | PARMAJ | 13.00 | . | 9.10 | 6.20 |
| **1992** | 10.82 | AAS | FECES | PARMAJ | 41.00 | . | UDL | 9.30 |
| **1993** | 0.92 | AAS | FECES | FICHYP | 293.91 | 41.57 | 10.11 | 2.160 |
| **1993** | 0.92 | AAS | FECES | FICHYP | 392.78 | 36.79 | UDL | 6.340 |
| **1993** | 0.92 | AAS | FECES | FICHYP | 220.93 | 17.34 | UDL | 3.710 |
| **1993** | 0.81 | AAS | FECES | FICHYP | 434.51 | 59.38 | 48.00 | 1.670 |
| **1993** | 0.81 | AAS | FECES | FICHYP | 278.11 | 45.80 | 17.56 | 4.790 |
| **1993** | 0.81 | AAS | FECES | FICHYP | 483.90 | 60.62 | UDL | 4.120 |
| **1993** | 0.81 | AAS | FECES | FICHYP | 503.79 | 53.56 | 24.14 | 4.390 |
| **1993** | 0.81 | AAS | FECES | FICHYP | 279.17 | 41.69 | 17.36 | 4.590 |
| **1993** | 0.81 | AAS | FECES | FICHYP | 775.41 | 94.16 | 27.66 | 3.500 |
| **1993** | 0.81 | AAS | FECES | FICHYP | 208.27 | 27.35 | 14.92 | 3.580 |
| **1993** | 0.96 | AAS | FECES | FICHYP | 304.97 | 57.54 | 11.89 | 2.180 |
| **1993** | 0.96 | AAS | FECES | FICHYP | 11900.28 | 154.59 | 16.31 | 2.840 |
| **1993** | 0.96 | AAS | FECES | FICHYP | 540.66 | 42.66 | UDL | 1.200 |
| **1993** | 1.20 | AAS | FECES | FICHYP | 138.99 | 29.93 | 8.58 | 2.400 |
| **1993** | 1.20 | AAS | FECES | FICHYP | 126.89 | UDL | UDL | 2.560 |
| **1993** | 1.20 | AAS | FECES | FICHYP | 230.06 | 28.03 | 14.49 | 4.220 |
| **1993** | 1.20 | AAS | FECES | FICHYP | 165.17 | 34.98 | 18.31 | 2.090 |
| **1993** | 1.20 | AAS | FECES | FICHYP | 185.06 | 31.07 | UDL | 4.930 |
| **1993** | 5.10 | AAS | FECES | FICHYP | 56.87 | 6.51 | UDL | 2.670 |
| **1993** | 5.10 | AAS | FECES | FICHYP | 108.70 | 11.54 | UDL | 2.040 |
| **1993** | 5.10 | AAS | FECES | FICHYP | 55.61 | 7.14 | UDL | 2.450 |
| **1993** | 5.10 | AAS | FECES | FICHYP | 47.56 | 9.45 | UDL | 1.770 |
| **1993** | 5.10 | AAS | FECES | FICHYP | 78.65 | 8.30 | UDL | 2.250 |
| **1993** | 5.10 | AAS | FECES | FICHYP | 66.48 | 5.57 | UDL | 2.240 |
| **1993** | 5.10 | AAS | FECES | FICHYP | 164.75 | 6.41 | UDL | 5.290 |
| **1993** | 10.78 | AAS | FECES | FICHYP | 46.64 | 5.43 | UDL | 3.360 |
| **1993** | 10.78 | AAS | FECES | FICHYP | 65.26 | 8.42 | 3.89 | 2.540 |
| **1993** | 10.78 | AAS | FECES | FICHYP | 82.95 | 6.72 | UDL | 2.870 |
| **1993** | 10.78 | AAS | FECES | FICHYP | 113.65 | 3.49 | UDL | 4.290 |
| **1993** | 10.78 | AAS | FECES | FICHYP | 45.56 | UDL | UDL | 5.800 |
| **1993** | 10.01 | AAS | FECES | FICHYP | 58.46 | UDL | UDL | 2.310 |
| **1993** | 10.01 | AAS | FECES | FICHYP | 73.39 | UDL | UDL | 3.380 |
| **1993** | 10.01 | AAS | FECES | FICHYP | 101.92 | UDL | UDL | 3.150 |
| **1993** | 10.01 | AAS | FECES | FICHYP | 85.29 | 4.58 | UDL | 1.790 |
| **1993** | 10.01 | AAS | FECES | FICHYP | 49.10 | UDL | UDL | 2.180 |
| **1993** | 10.01 | AAS | FECES | FICHYP | 61.81 | UDL | UDL | 2.700 |
| **1993** | 4.38 | AAS | FECES | FICHYP | 80.08 | UDL | UDL | 1.850 |
| **1993** | 4.38 | AAS | FECES | FICHYP | 100.60 | 7.62 | UDL | 3.450 |
| **1993** | 4.38 | AAS | FECES | FICHYP | 169.17 | UDL | UDL | 3.130 |
| **1993** | 4.38 | AAS | FECES | FICHYP | 73.84 | 7.14 | UDL | 3.250 |
| **1993** | 4.38 | AAS | FECES | FICHYP | 66.38 | 10.28 | UDL | 2.670 |
| **1993** | 4.38 | AAS | FECES | FICHYP | 129.97 | 9.39 | UDL | 3.450 |
| **1993** | 10.82 | AAS | FECES | FICHYP | 79.54 | UDL | UDL | 3.910 |
| **1993** | 10.82 | AAS | FECES | FICHYP | 46.59 | UDL | UDL | 1.660 |
| **1993** | 10.82 | AAS | FECES | FICHYP | 68.04 | 2.34 | UDL | 3.010 |
| **1993** | 10.82 | AAS | FECES | FICHYP | 114.29 | 0.00 | UDL | 0.000 |
| **1993** | 10.82 | AAS | FECES | FICHYP | 57.16 | 3.52 | UDL | 3.230 |
| **1993** | 0.92 | AAS | FECES | PARMAJ | 171.57 | 26.53 | 12.10 | 1.710 |
| **1993** | 0.92 | AAS | FECES | PARMAJ | 246.60 | 47.36 | 27.01 | 2.590 |
| **1993** | 0.92 | AAS | FECES | PARMAJ | 170.64 | 13.01 | UDL | 7.590 |
| **1993** | 0.92 | AAS | FECES | PARMAJ | 207.70 | 29.57 | 39.62 | 2.050 |
| **1993** | 0.81 | AAS | FECES | PARMAJ | 338.69 | 46.91 | UDL | 5.990 |
| **1993** | 0.81 | AAS | FECES | PARMAJ | 250.54 | 36.96 | 22.27 | 4.790 |
| **1993** | 0.96 | AAS | FECES | PARMAJ | 68.64 | 18.18 | 14.42 | 1.750 |
| **1993** | 1.20 | AAS | FECES | PARMAJ | 582.75 | 71.03 | UDL | 7.380 |
| **1993** | 5.10 | AAS | FECES | PARMAJ | 29.99 | 9.35 | UDL | 0.790 |
| **1993** | 5.10 | AAS | FECES | PARMAJ | 95.38 | 13.42 | UDL | 1.700 |
| **1993** | 5.10 | AAS | FECES | PARMAJ | 57.09 | 6.13 | UDL | 1.510 |
| **1993** | 5.10 | AAS | FECES | PARMAJ | 63.91 | 8.48 | UDL | 0.850 |
| **1993** | 5.10 | AAS | FECES | PARMAJ | 115.23 | 5.77 | UDL | 3.790 |
| **1993** | 5.10 | AAS | FECES | PARMAJ | 55.63 | UDL | UDL | 2.170 |
| **1993** | 5.10 | AAS | FECES | PARMAJ | 60.75 | 5.98 | UDL | 3.080 |
| **1993** | 5.10 | AAS | FECES | PARMAJ | 43.47 | UDL | UDL | 1.540 |
| **1993** | 10.78 | AAS | FECES | PARMAJ | 48.15 | 8.85 | UDL | 1.380 |
| **1993** | 10.78 | AAS | FECES | PARMAJ | 103.94 | 9.30 | UDL | 1.600 |
| **1993** | 10.78 | AAS | FECES | PARMAJ | 27.96 | UDL | UDL | 1.020 |
| **1993** | 10.78 | AAS | FECES | PARMAJ | 80.04 | 12.00 | UDL | 8.010 |
| **1993** | 10.01 | AAS | FECES | PARMAJ | 54.40 | 5.24 | 11.63 | 2.030 |
| **1993** | 10.01 | AAS | FECES | PARMAJ | 61.50 | 9.07 | 10.87 | 2.030 |
| **1993** | 10.01 | AAS | FECES | PARMAJ | 81.23 | UDL | UDL | 4.630 |
| **1993** | 4.38 | AAS | FECES | PARMAJ | 25.47 | 7.97 | UDL | 0.610 |
| **1993** | 4.38 | AAS | FECES | PARMAJ | 50.53 | UDL | UDL | 1.150 |
| **1993** | 4.38 | AAS | FECES | PARMAJ | 74.80 | UDL | UDL | 1.240 |
| **1993** | 4.38 | AAS | FECES | PARMAJ | 158.76 | 24.50 | UDL | 1.960 |
| **1993** | 4.38 | AAS | FECES | PARMAJ | 41.61 | 8.27 | UDL | 0.880 |
| **1993** | 4.38 | AAS | FECES | PARMAJ | 60.96 | UDL | UDL | 1.470 |
| **1993** | 4.38 | AAS | FECES | PARMAJ | 85.70 | 12.13 | UDL | 3.130 |
| **1993** | 10.82 | AAS | FECES | PARMAJ | 27.86 | 4.61 | UDL | 1.310 |
| **1993** | 10.82 | AAS | FECES | PARMAJ | 32.92 | 5.63 | 13.55 | 1.150 |
| **1993** | 10.82 | AAS | FECES | PARMAJ | 54.17 | 4.16 | UDL | 1.500 |
| **1993** | 10.82 | AAS | FECES | PARMAJ | 22.84 | 4.84 | UDL | 1.750 |
| **1993** | 10.82 | AAS | FECES | PARMAJ | 44.36 | UDL | UDL | 1.630 |
| **1993** |  | AAS | PMWINT | . | 346.58 | 55.61 | 30.86 | 2.310 |
| **1993** |  | AAS | PMWINT | . | 301.78 | 67.00 | 24.98 | 2.060 |
| **1993** |  | AAS | PMWINT | . | 373.14 | 66.00 | 24.69 | 2.310 |
| **1993** |  | AAS | PMWINT | . | 355.37 | 54.35 | 29.07 | 2.290 |
| **1993** |  | AAS | PMWINT | . | 331.82 | 56.32 | 23.14 | 2.300 |
| **1994** | 0.83 | AAS | FECES | FICHYP | 153.47 | 18.89 | 7.66 | 2.49 |
| **1994** | 0.83 | AAS | FECES | FICHYP | 187.77 | 28.55 | 9.92 | 3.71 |
| **1994** | 0.81 | AAS | FECES | FICHYP | 353.02 | 43.09 | 14.30 | 3.97 |
| **1994** | 0.85 | AAS | FECES | FICHYP | 410.36 | 64.22 | 33.07 | 5.85 |
| **1994** | 0.87 | AAS | FECES | FICHYP | 358.99 | 43.15 | 22.28 | 6.56 |
| **1994** | 0.87 | AAS | FECES | FICHYP | 105.31 | 14.81 | UDL | 3.70 |
| **1994** | 0.64 | AAS | FECES | FICHYP | 491.85 | 63.26 | 15.89 | 5.43 |
| **1994** | 0.74 | AAS | FECES | FICHYP | 389.70 | 49.72 | 14.90 | 6.81 |
| **1994** | 0.80 | AAS | FECES | FICHYP | 301.00 | 50.02 | 10.58 | 5.14 |
| **1994** | 0.66 | AAS | FECES | FICHYP | 240.08 | 33.41 | 8.43 | 5.06 |
| **1994** | 0.66 | AAS | FECES | FICHYP | 213.82 | 31.08 | UDL | 6.34 |
| **1994** | 0.77 | AAS | FECES | FICHYP | 297.89 | 54.40 | 11.84 | 3.18 |
| **1994** | 0.80 | AAS | FECES | FICHYP | 159.14 | 26.78 | UDL | 4.53 |
| **1994** | 0.78 | AAS | FECES | FICHYP | 241.23 | 53.28 | 8.16 | 4.94 |
| **1994** | 0.84 | AAS | FECES | FICHYP | 137.31 | 29.02 | UDL | 2.76 |
| **1994** | 0.74 | AAS | FECES | FICHYP | 217.19 | 24.24 | UDL | 3.27 |
| **1994** | 0.81 | AAS | FECES | FICHYP | 304.06 | 38.59 | UDL | 3.18 |
| **1999** | 0.99 | AAS | FECES | PARMAJ | 308.74 | . | 5.63 | . |
| **1999** | 1.02 | AAS | FECES | PARMAJ | 271.78 | . | 9.35 | . |
| **1999** | 1.12 | AAS | FECES | PARMAJ | 115.10 | . | 5.95 | . |
| **1999** | 1.02 | AAS | FECES | PARMAJ | 145.37 | . | 5.42 | . |
| **1999** | 0.99 | AAS | FECES | PARMAJ | 215.48 | . | 12.22 | . |
| **1999** | 0.91 | AAS | FECES | PARMAJ | 261.72 | . | 11.13 | . |
| **1999** | 0.68 | AAS | FECES | PARMAJ | 324.14 | . | 11.03 | . |
| **1999** | 0.91 | AAS | FECES | PARMAJ | 297.80 | . | 11.20 | . |
| **1999** | 0.88 | AAS | FECES | PARMAJ | 928.67 | . | 20.05 | . |
| **1999** | 0.66 | AAS | FECES | PARMAJ | 199.02 | . | 5.85 | . |
| **1999** | 1.28 | AAS | FECES | PARMAJ | 230.69 | . | 3.80 | . |
| **1999** | 1.22 | AAS | FECES | PARMAJ | 94.83 | . | 3.64 | . |
| **1999** | 1.12 | AAS | FECES | PARMAJ | 85.49 | . | 2.55 | . |
| **1999** | 0.95 | AAS | FECES | PARMAJ | 126.17 | . | 6.19 | . |
| **1999** | 0.87 | AAS | FECES | PARMAJ | 311.74 | . | 6.04 | . |
| **1999** | 10.30 | AAS | FECES | PARMAJ | 108.65 | . | 0.94 | . |
| **1999** | 10.05 | AAS | FECES | PARMAJ | 124.80 | . | 2.08 | . |
| **1999** | 10.18 | AAS | FECES | PARMAJ | 60.91 | . | 3.48 | . |
| **1999** | 10.23 | AAS | FECES | PARMAJ | 43.01 | . | 3.30 | . |
| **1999** | 10.15 | AAS | FECES | PARMAJ | 47.59 | . | 0.43 | . |
| **1999** | 9.92 | AAS | FECES | PARMAJ | 123.59 | . | 1.19 | . |
| **1999** | 9.89 | AAS | FECES | PARMAJ | 91.77 | . | 2.29 | . |
| **1999** | 9.92 | AAS | FECES | PARMAJ | 99.74 | . | 1.81 | . |
| **1999** | 10.01 | AAS | FECES | PARMAJ | 43.69 | . | 1.98 | . |
| **1999** | 4.17 | AAS | FECES | PARMAJ | 106.23 | . | 2.62 | . |
| **1999** | 4.21 | AAS | FECES | PARMAJ | 51.55 | . | 3.35 | . |
| **1999** | 4.08 | AAS | FECES | PARMAJ | 87.40 | . | 1.39 | . |
| **1999** | 11.00 | AAS | FECES | PARMAJ | 49.68 | . | 0.89 | . |
| **1999** | 11.05 | AAS | FECES | PARMAJ | 103.26 | . | 0.77 | . |
| **1999** | 11.08 | AAS | FECES | PARMAJ | 269.50 | . | 3.75 | . |
| **1999** | 10.83 | AAS | FECES | PARMAJ | 58.51 | . | 2.59 | . |
| **1999** | 10.74 | AAS | FECES | PARMAJ | 123.20 | . | 0.55 | . |
| **1999** | 10.68 | AAS | FECES | PARMAJ | 80.59 | . | 1.72 | . |
| **1999** | 10.59 | AAS | FECES | PARMAJ | 74.97 | . | 0.65 | . |
| **1999** | 10.59 | AAS | FECES | PARMAJ | 31.76 | . | 1.25 | . |
| **1999** | 5.32 | AAS | FECES | PARMAJ | 66.70 | . | 1.13 | . |
| **1999** | 5.56 | AAS | FECES | PARMAJ | 79.08 | . | 1.42 | . |
| **1999** | 5.26 | AAS | FECES | PARMAJ | 167.40 | . | 2.32 | . |
| **1999** | 5.35 | AAS | FECES | PARMAJ | 63.92 | . | 2.07 | . |
| **1999** | 5.20 | AAS | FECES | PARMAJ | 95.68 | . | 1.16 | . |
| **1999** | 1.67 | AAS | FECES | PARMAJ | 172.92 | . | 3.28 | . |
| **1999** | 1.66 | AAS | FECES | PARMAJ | 154.41 | . | 1.71 | . |
| **1999** | 1.71 | AAS | FECES | PARMAJ | 1093.99 | . | 2.50 | . |
| **1999** | 1.92 | AAS | FECES | PARMAJ | 103.37 | . | 18.92 | . |
| **1999** | 1.93 | AAS | FECES | PARMAJ | 63.74 | . | 2.61 | . |
| **1999** | 1.93 | AAS | FECES | PARMAJ | 166.12 | . | 1.52 | . |
| **1999** | 1.61 | AAS | FECES | PARMAJ | 461.68 | . | 9.42 | . |
| **1999** | 1.58 | AAS | FECES | PARMAJ | 75.30 | . | 3.29 | . |
| **1999** | 1.54 | AAS | FECES | PARMAJ | 110.30 | . | 3.13 | . |
| **1999** | 1.73 | AAS | FECES | PARMAJ | 175.70 | . | 4.17 | . |
| **1999** | 1.65 | AAS | FECES | PARMAJ | 75.80 | . | 16.34 | . |
| **1999** | 1.53 | AAS | FECES | PARMAJ | 151.75 | . | 14.64 | . |
| **1999** | 0.56 | AAS | FECES | PARMAJ | 495.04 | . | 16.82 | . |
| **1999** |  | AAS | PMWINT | . | 446.45 | . | 19.82 | . |
| **1999** |  | AAS | PMWINT | . | 409.04 | . | 25.13 | . |
| **1999** |  | AAS | PMWINT | . | 430.46 | . | 18.29 | . |
| **1999** |  | AAS | PMWINT | . | 413.56 | . | 19.77 | . |
| **1999** |  | AAS | PMWINT | . | 421.92 | . | 26.85 | . |
| **1999** |  | AAS | PMWINT | . | 402.94 | . | 23.63 | . |
| **2002** | 1.03 | ICP | FECES | FICHYP | 546.27 | 125.41 | 9.50 | 5.89 |
| **2002** | 0.99 | ICP | FECES | FICHYP | 467.32 | 94.63 | 4.88 | 3.97 |
| **2002** | 0.87 | ICP | FECES | FICHYP | 423.00 | 59.19 | 7.54 | 5.33 |
| **2002** | 1.29 | ICP | FECES | FICHYP | 124.13 | 28.24 | 1.76 | 1.68 |
| **2002** | 1.24 | ICP | FECES | FICHYP | 250.23 | 44.66 | 7.65 | 2.27 |
| **2002** | 0.93 | ICP | FECES | FICHYP | 274.08 | 75.43 | 6.24 | 7.93 |
| **2002** | 5.04 | ICP | FECES | FICHYP | 212.54 | 9.86 | 1.65 | 6.69 |
| **2002** | 5.08 | ICP | FECES | FICHYP | 36.79 | 4.79 | 2.11 | 0.78 |
| **2002** | 5.05 | ICP | FECES | FICHYP | 145.79 | 9.13 | 2.18 | 3.15 |
| **2002** | 11.22 | ICP | FECES | FICHYP | 80.75 | 6.43 | 1.31 | 1.84 |
| **2002** | 11.04 | ICP | FECES | FICHYP | 75.64 | 4.13 | 2.09 | 3.50 |
| **2002** | 9.99 | ICP | FECES | FICHYP | 98.24 | 2.63 | 0.58 | 2.90 |
| **2002** | 10.21 | ICP | FECES | FICHYP | 50.48 | 3.88 | 1.58 | 2.89 |
| **2002** | 11.03 | ICP | FECES | FICHYP | 71.40 | 5.68 | 0.92 | 2.05 |
| **2002** | 10.65 | ICP | FECES | FICHYP | 47.61 | 3.11 | 0.90 | 2.41 |
| **2002** | 10.59 | ICP | FECES | FICHYP | 50.04 | 3.15 | 2.20 | 4.18 |
| **2002** | 5.36 | ICP | FECES | FICHYP | 76.60 | 6.67 | 1.81 | 3.46 |
| **2002** | 5.53 | ICP | FECES | FICHYP | 41.89 | 2.79 | 0.96 | 1.58 |
| **2002** | 5.46 | ICP | FECES | FICHYP | 70.67 | 2.80 | 1.56 | 3.14 |
| **2002** | 1.88 | ICP | FECES | FICHYP | 204.64 | 28.38 | 38.44 | 4.45 |
| **2002** | 1.80 | ICP | FECES | FICHYP | 150.79 | 33.87 | 3.56 | 2.75 |
| **2002** | 1.65 | ICP | FECES | FICHYP | 139.35 | 23.87 | 2.24 | 3.23 |
| **2002** | 1.73 | ICP | FECES | FICHYP | 263.87 | 57.23 | 9.43 | 7.51 |
| **2002** | 1.07 | ICP | FECES | FICHYP | 212.88 | 39.42 | 7.75 | 4.12 |
| **2002** | 1.20 | ICP | FECES | FICHYP | 236.67 | 22.72 | 1.74 | 5.22 |
| **2002** | 1.15 | ICP | FECES | FICHYP | 143.53 | 29.95 | 18.07 | 2.57 |
| **2002** | 0.98 | ICP | FECES | PARMAJ | 341.42 | 188.54 | 5.76 | 1.56 |
| **2002** | 1.07 | ICP | FECES | PARMAJ | 136.90 | 33.93 | 3.75 | 2.84 |
| **2002** | 0.97 | ICP | FECES | PARMAJ | 274.93 | 218.62 | 8.69 | 4.04 |
| **2002** | 1.03 | ICP | FECES | PARMAJ | 184.69 | 38.51 | 9.47 | 3.07 |
| **2002** | 0.90 | ICP | FECES | PARMAJ | 122.99 | 30.05 | 10.22 | 2.71 |
| **2002** | 1.09 | ICP | FECES | PARMAJ | 136.48 | 27.40 | 4.08 | 3.41 |
| **2002** | 5.09 | ICP | FECES | PARMAJ | 67.83 | 9.08 | 3.65 | 0.70 |
| **2002** | 4.93 | ICP | FECES | PARMAJ | 57.23 | 7.07 | 1.37 | 2.26 |
| **2002** | 4.97 | ICP | FECES | PARMAJ | 88.60 | 5.55 | 9.25 | 1.82 |
| **2002** | 11.16 | ICP | FECES | PARMAJ | 32.71 | 11.03 | 3.80 | 0.68 |
| **2002** | 11.03 | ICP | FECES | PARMAJ | 49.36 | 7.39 | 1.48 | 0.68 |
| **2002** | 10.13 | ICP | FECES | PARMAJ | 62.65 | 6.77 | 0.52 | 0.58 |
| **2002** | 10.20 | ICP | FECES | PARMAJ | 36.20 | 6.29 | 1.16 | 0.65 |
| **2002** | 10.13 | ICP | FECES | PARMAJ | 47.04 | 2.46 | 0.86 | 1.59 |
| **2002** | 11.13 | ICP | FECES | PARMAJ | 43.61 | 5.52 | 1.43 | 0.70 |
| **2002** | 10.97 | ICP | FECES | PARMAJ | 47.19 | 10.06 | 3.64 | 1.04 |
| **2002** | 10.73 | ICP | FECES | PARMAJ | 54.57 | 2.40 | 1.00 | 1.29 |
| **2002** | 5.26 | ICP | FECES | PARMAJ | 109.38 | 6.23 | 3.86 | 2.25 |
| **2002** | 5.20 | ICP | FECES | PARMAJ | 80.80 | 9.05 | 5.89 | 0.95 |
| **2002** | 1.83 | ICP | FECES | PARMAJ | 183.43 | 54.55 | 6.74 | 1.37 |
| **2002** | 1.77 | ICP | FECES | PARMAJ | 119.37 | 17.49 | 1.29 | 0.99 |
| **2002** | 1.62 | ICP | FECES | PARMAJ | 88.97 | 16.25 | 1.19 | 1.56 |
| **2002** | 1.57 | ICP | FECES | PARMAJ | 234.81 | 33.55 | 6.70 | 3.46 |
| **2002** | 1.63 | ICP | FECES | PARMAJ | 110.90 | 20.15 | 2.32 | 2.84 |
| **2002** | 1.21 | ICP | FECES | PARMAJ | 134.74 | 22.50 | 1.94 | 1.49 |
| **2002** | 1.15 | ICP | FECES | PARMAJ | 206.99 | 37.70 | 18.28 | 4.13 |
| **2004** | 1.08 | ICP | FECES | PARMAJ | 759.85 | 111.53 | 24.76 | 4.84 |
| **2004** | 1.02 | ICP | FECES | PARMAJ | 219.45 | 53.90 | 4.73 | 3.08 |
| **2004** | 0.89 | ICP | FECES | PARMAJ | 351.74 | 68.72 | 4.02 | 6.43 |
| **2004** | 1.00 | ICP | FECES | PARMAJ | 247.65 | 55.67 | 4.88 | 2.42 |
| **2004** | 0.95 | ICP | FECES | PARMAJ | 480.10 | 78.32 | 7.66 | 14.08 |
| **2004** | 0.84 | ICP | FECES | PARMAJ | 407.99 | 75.44 | 7.15 | 2.52 |
| **2004** | 0.91 | ICP | FECES | PARMAJ | 513.31 | 49.00 | 7.34 | 7.03 |
| **2004** | 1.31 | ICP | FECES | PARMAJ | 144.49 | 24.48 | 3.62 | 2.94 |
| **2004** | 1.25 | ICP | FECES | PARMAJ | 78.01 | 13.40 | 2.22 | 1.97 |
| **2004** | 1.09 | ICP | FECES | PARMAJ | 98.26 | 10.56 | 1.38 | 1.97 |
| **2004** | 1.03 | ICP | FECES | PARMAJ | 111.39 | 19.25 | 2.68 | 1.02 |
| **2004** | 0.96 | ICP | FECES | PARMAJ | 143.69 | 28.68 | 3.41 | 1.07 |
| **2004** | 5.22 | ICP | FECES | PARMAJ | 108.04 | 10.69 | 2.29 | 1.17 |
| **2004** | 5.11 | ICP | FECES | PARMAJ | 233.86 | 9.29 | 2.40 | 2.67 |
| **2004** | 5.16 | ICP | FECES | PARMAJ | 123.74 | 15.87 | 3.71 | 1.85 |
| **2004** | 5.08 | ICP | FECES | PARMAJ | 73.44 | 6.06 | 2.10 | 1.02 |
| **2004** | 5.04 | ICP | FECES | PARMAJ | 267.52 | 3.85 | 0.92 | 0.51 |
| **2004** | 5.00 | ICP | FECES | PARMAJ | 66.06 | 2.37 | 0.53 | 1.21 |
| **2004** | 4.93 | ICP | FECES | PARMAJ | 81.96 | 3.83 | 0.88 | 1.79 |
| **2004** | 4.84 | ICP | FECES | PARMAJ | 205.79 | 9.47 | 4.02 | 3.59 |
| **2004** | 11.07 | ICP | FECES | PARMAJ | 73.06 | 6.03 | 1.60 | 1.28 |
| **2004** | 11.16 | ICP | FECES | PARMAJ | 38.03 | 2.49 | 1.21 | 0.38 |
| **2004** | 11.11 | ICP | FECES | PARMAJ | 166.89 | 7.81 | 2.01 | 1.62 |
| **2004** | 11.10 | ICP | FECES | PARMAJ | 112.49 | 13.90 | 4.34 | 1.55 |
| **2004** | 11.00 | ICP | FECES | PARMAJ | 50.45 | 5.98 | 1.65 | 0.55 |
| **2004** | 10.29 | ICP | FECES | PARMAJ | 51.17 | 5.31 | 1.47 | 1.01 |
| **2004** | 10.08 | ICP | FECES | PARMAJ | 146.45 | 14.48 | 4.77 | 3.02 |
| **2004** | 10.26 | ICP | FECES | PARMAJ | 62.59 | 2.87 | 1.24 | 2.83 |
| **2004** | 10.07 | ICP | FECES | PARMAJ | 130.93 | 4.35 | 1.17 | 1.43 |
| **2004** | 9.91 | ICP | FECES | PARMAJ | 96.72 | 6.04 | 1.01 | 1.04 |
| **2004** | 9.79 | ICP | FECES | PARMAJ | 77.83 | 7.55 | 1.21 | 1.18 |
| **2004** | 9.92 | ICP | FECES | PARMAJ | 101.23 | 22.19 | 4.38 | 2.16 |
| **2004** | 10.95 | ICP | FECES | PARMAJ | 122.73 | 2.64 | 0.92 | 2.53 |
| **2004** | 11.05 | ICP | FECES | PARMAJ | 58.48 | 21.19 | 6.89 | 0.63 |
| **2004** | 11.13 | ICP | FECES | PARMAJ | 52.93 | 3.23 | 1.82 | 0.79 |
| **2004** | 10.90 | ICP | FECES | PARMAJ | 121.53 | 3.72 | 1.40 | 1.77 |
| **2004** | 10.72 | ICP | FECES | PARMAJ | 72.58 | 3.92 | 3.04 | 1.95 |
| **2004** | 10.55 | ICP | FECES | PARMAJ | 80.23 | 8.84 | 2.01 | 1.01 |
| **2004** | 5.54 | ICP | FECES | PARMAJ | 54.89 | 14.15 | 16.07 | 0.95 |
| **2004** | 5.33 | ICP | FECES | PARMAJ | 114.85 | 6.08 | 2.29 | 2.78 |
| **2004** | 5.29 | ICP | FECES | PARMAJ | 62.46 | 5.57 | 3.12 | 0.95 |
| **2004** | 5.26 | ICP | FECES | PARMAJ | 80.90 | 4.60 | 1.13 | 1.74 |
| **2004** | 5.28 | ICP | FECES | PARMAJ | 162.81 | 5.57 | 2.91 | 3.35 |
| **2004** | 5.36 | ICP | FECES | PARMAJ | 114.30 | 6.52 | 4.55 | 1.48 |
| **2004** | 5.48 | ICP | FECES | PARMAJ | 181.66 | 7.55 | 2.56 | 4.92 |
| **2004** | 5.38 | ICP | FECES | PARMAJ | 185.88 | 7.09 | 1.73 | 3.27 |
| **2004** | 5.26 | ICP | FECES | PARMAJ | 91.72 | 6.22 | 5.25 | 2.92 |
| **2004** | 1.67 | ICP | FECES | PARMAJ | 257.28 | 31.09 | 8.38 | 2.68 |
| **2004** | 1.84 | ICP | FECES | PARMAJ | 232.49 | 26.86 | 3.70 | 3.93 |
| **2004** | 1.70 | ICP | FECES | PARMAJ | 156.65 | 29.56 | 15.66 | 2.00 |
| **2004** | 1.65 | ICP | FECES | PARMAJ | 109.00 | 18.93 | 3.47 | 1.39 |
| **2004** | 1.53 | ICP | FECES | PARMAJ | 182.79 | 34.90 | 4.71 | 2.67 |
| **2004** | 1.03 | ICP | FECES | PARMAJ | 246.58 | 32.41 | 6.84 | 6.42 |
| **2004** | 1.12 | ICP | FECES | PARMAJ | 179.53 | 15.25 | 2.47 | 2.96 |
| **2004** | 1.08 | ICP | FECES | PARMAJ | 213.87 | 35.13 | 2.79 | 3.42 |
| **2004** | 1.14 | ICP | FECES | PARMAJ | 202.60 | 17.49 | 3.16 | 3.52 |
| **2004** | 1.22 | ICP | FECES | PARMAJ | 174.98 | 33.88 | 1.96 | 3.54 |
| **2004** | 1.37 | ICP | FECES | PARMAJ | 88.58 | 26.28 | 3.11 | 0.70 |
| **2004** | 1.28 | ICP | FECES | PARMAJ | 130.99 | 13.99 | 2.71 | 1.93 |
| **2004** | 1.20 | ICP | FECES | PARMAJ | 317.39 | 19.26 | 2.21 | 14.16 |
| **2004** | 1.23 | ICP | FECES | PARMAJ | 215.34 | 24.32 | 2.88 | 4.66 |
| **2005** | 1.04 | ICP | FECES | PARMAJ | 447.32 | 49.33 | 4.78 | 9.30 |
| **2005** | 1.03 | ICP | FECES | PARMAJ | 385.24 | 63.66 | 24.25 | 6.62 |
| **2005** | 1.06 | ICP | FECES | PARMAJ | 195.01 | 32.26 | 5.72 | 2.58 |
| **2005** | 0.98 | ICP | FECES | PARMAJ | 772.20 | 92.77 | 32.33 | 9.84 |
| **2005** | 0.91 | ICP | FECES | PARMAJ | 147.90 | 25.17 | 5.83 | 2.85 |
| **2005** | 0.92 | ICP | FECES | PARMAJ | 375.35 | 32.36 | 5.63 | 5.30 |
| **2005** | 0.93 | ICP | FECES | PARMAJ | 482.00 | 35.70 | 45.96 | 3.82 |
| **2005** | 1.35 | ICP | FECES | PARMAJ | 110.07 | 11.33 | 1.59 | 2.78 |
| **2005** | 1.26 | ICP | FECES | PARMAJ | 137.12 | 17.92 | 7.41 | 3.03 |
| **2005** | 1.12 | ICP | FECES | PARMAJ | 83.13 | 11.23 | 1.41 | 1.57 |
| **2005** | 0.89 | ICP | FECES | PARMAJ | 310.41 | 46.49 | 9.79 | 7.63 |
| **2005** | 5.26 | ICP | FECES | PARMAJ | 66.56 | 5.16 | 2.75 | 1.86 |
| **2005** | 5.22 | ICP | FECES | PARMAJ | 62.91 | 3.71 | 0.80 | 0.76 |
| **2005** | 5.20 | ICP | FECES | PARMAJ | 68.72 | 3.43 | 0.96 | 0.54 |
| **2005** | 5.04 | ICP | FECES | PARMAJ | 100.57 | 9.17 | 5.91 | 2.23 |
| **2005** | 5.11 | ICP | FECES | PARMAJ | 82.99 | 4.68 | 1.88 | 0.59 |
| **2005** | 5.00 | ICP | FECES | PARMAJ | 170.09 | 3.56 | 1.47 | 5.19 |
| **2005** | 4.95 | ICP | FECES | PARMAJ | 301.00 | 28.05 | 4.34 | 5.62 |
| **2005** | 4.97 | ICP | FECES | PARMAJ | 64.32 | 7.43 | 5.10 | 0.64 |
| **2005** | 11.18 | ICP | FECES | PARMAJ | 40.86 | 2.94 | 1.33 | 0.49 |
| **2005** | 11.16 | ICP | FECES | PARMAJ | 32.92 | 1.20 | 1.75 | 0.85 |
| **2005** | 11.12 | ICP | FECES | PARMAJ | 21.86 | 2.49 | 1.90 | 0.32 |
| **2005** | 11.11 | ICP | FECES | PARMAJ | 42.49 | 4.68 | 2.38 | 0.86 |
| **2005** | 11.20 | ICP | FECES | PARMAJ | 147.48 | 1.45 | 0.45 | 2.37 |
| **2005** | 11.21 | ICP | FECES | PARMAJ | 97.25 | 1.32 | 0.53 | 1.65 |
| **2005** | 11.09 | ICP | FECES | PARMAJ | 113.56 | 3.64 | 3.32 | 2.54 |
| **2005** | 11.08 | ICP | FECES | PARMAJ | 39.85 | 2.05 | 0.84 | 0.65 |
| **2005** | 10.29 | ICP | FECES | PARMAJ | 51.88 | 3.31 | 1.97 | 1.36 |
| **2005** | 10.21 | ICP | FECES | PARMAJ | 45.99 | 3.43 | 3.91 | 1.44 |
| **2005** | 9.99 | ICP | FECES | PARMAJ | 95.18 | 2.01 | 0.97 | 2.93 |
| **2005** | 10.13 | ICP | FECES | PARMAJ | 73.17 | 7.24 | 7.88 | 0.89 |
| **2005** | 10.24 | ICP | FECES | PARMAJ | 59.06 | 4.12 | 2.71 | 0.91 |
| **2005** | 10.20 | ICP | FECES | PARMAJ | 59.52 | 2.44 | 1.07 | 0.97 |
| **2005** | 10.09 | ICP | FECES | PARMAJ | 72.46 | 2.91 | 1.81 | 1.31 |
| **2005** | 10.07 | ICP | FECES | PARMAJ | 50.00 | 3.43 | 4.67 | 1.01 |
| **2005** | 9.80 | ICP | FECES | PARMAJ | 152.12 | 2.46 | 2.08 | 3.54 |
| **2005** | 9.86 | ICP | FECES | PARMAJ | 72.16 | 2.19 | 0.84 | 1.33 |
| **2005** | 11.01 | ICP | FECES | PARMAJ | 40.40 | 2.11 | 0.63 | 1.95 |
| **2005** | 11.08 | ICP | FECES | PARMAJ | 49.39 | 1.34 | 2.00 | 1.39 |
| **2005** | 11.18 | ICP | FECES | PARMAJ | 31.76 | 1.65 | 0.73 | 0.78 |
| **2005** | 11.03 | ICP | FECES | PARMAJ | 62.69 | 7.28 | 4.57 | 2.57 |
| **2005** | 11.01 | ICP | FECES | PARMAJ | 47.23 | 2.29 | 1.28 | 1.65 |
| **2005** | 10.92 | ICP | FECES | PARMAJ | 33.69 | 3.54 | 5.44 | 0.49 |
| **2005** | 10.87 | ICP | FECES | PARMAJ | 106.53 | 6.27 | 2.74 | 2.98 |
| **2005** | 10.68 | ICP | FECES | PARMAJ | 59.36 | 3.14 | 2.24 | 0.95 |
| **2005** | 10.65 | ICP | FECES | PARMAJ | 32.85 | 1.25 | 0.94 | 2.46 |
| **2005** | 10.59 | ICP | FECES | PARMAJ | 62.54 | 5.22 | 1.58 | 1.21 |
| **2005** | 10.55 | ICP | FECES | PARMAJ | 41.92 | 3.00 | 4.85 | 0.87 |
| **2005** | 5.32 | ICP | FECES | PARMAJ | 53.11 | 4.34 | 1.22 | 1.89 |
| **2005** | 5.44 | ICP | FECES | PARMAJ | 28.61 | 5.04 | 1.62 | 0.76 |
| **2005** | 5.54 | ICP | FECES | PARMAJ | 58.83 | 2.32 | 1.41 | 1.42 |
| **2005** | 5.60 | ICP | FECES | PARMAJ | 33.84 | 2.16 | 1.77 | 0.94 |
| **2005** | 5.56 | ICP | FECES | PARMAJ | 95.76 | 3.24 | 1.00 | 1.77 |
| **2005** | 5.29 | ICP | FECES | PARMAJ | 118.01 | 2.77 | 21.17 | 2.39 |
| **2005** | 5.39 | ICP | FECES | PARMAJ | 58.60 | 2.15 | 2.91 | 1.79 |
| **2005** | 5.51 | ICP | FECES | PARMAJ | 151.12 | 1.09 | 0.73 | 4.51 |
| **2005** | 5.35 | ICP | FECES | PARMAJ | 48.27 | 3.54 | 2.21 | 1.27 |
| **2005** | 5.20 | ICP | FECES | PARMAJ | 31.14 | 1.01 | 1.11 | 0.32 |
| **2005** | 1.76 | ICP | FECES | PARMAJ | 170.61 | 28.66 | 119.91 | 2.56 |
| **2005** | 1.75 | ICP | FECES | PARMAJ | 147.38 | 29.05 | 4.66 | 2.12 |
| **2005** | 1.88 | ICP | FECES | PARMAJ | 183.47 | 13.33 | 8.05 | 3.04 |
| **2005** | 1.80 | ICP | FECES | PARMAJ | 123.92 | 12.67 | 10.13 | 2.55 |
| **2005** | 1.86 | ICP | FECES | PARMAJ | 226.96 | 14.61 | 580.00 | 1.11 |
| **2005** | 1.61 | ICP | FECES | PARMAJ | 129.55 | 22.11 | 3.51 | 2.98 |
| **2005** | 1.63 | ICP | FECES | PARMAJ | 135.46 | 17.45 | 4.09 | 1.92 |
| **2005** | 1.67 | ICP | FECES | PARMAJ | 150.44 | 17.58 | 3.12 | 2.28 |
| **2005** | 1.72 | ICP | FECES | PARMAJ | 74.29 | 8.94 | 3.09 | 1.96 |
| **2005** | 1.73 | ICP | FECES | PARMAJ | 183.51 | 14.89 | 3.17 | 3.89 |
| **2005** | 1.63 | ICP | FECES | PARMAJ | 94.95 | 8.75 | 2.99 | 6.69 |
| **2005** | 1.53 | ICP | FECES | PARMAJ | 157.61 | 14.94 | 10.45 | 5.59 |
| **2005** | 1.03 | ICP | FECES | PARMAJ | 175.56 | 22.46 | 3.91 | 1.57 |
| **2005** | 1.08 | ICP | FECES | PARMAJ | 117.31 | 11.86 | 1.69 | 3.33 |
| **2005** | 1.12 | ICP | FECES | PARMAJ | 237.30 | 21.28 | 5.94 | 5.56 |
| **2005** | 1.21 | ICP | FECES | PARMAJ | 223.07 | 22.19 | 4.10 | 3.98 |
| **2005** | 1.21 | ICP | FECES | PARMAJ | 96.21 | 8.61 | 3.16 | 1.56 |
| **2005** | 1.16 | ICP | FECES | PARMAJ | 164.44 | 20.47 | 3.29 | 2.63 |
| **2005** | 1.17 | ICP | FECES | PARMAJ | 209.14 | 22.21 | 3.99 | 7.26 |
| **2005** | 1.37 | ICP | FECES | PARMAJ | 79.51 | 9.50 | 1.80 | 1.10 |
| **2005** | 1.35 | ICP | FECES | PARMAJ | 185.27 | 14.25 | 4.72 | 6.96 |
| **2005** | 1.22 | ICP | FECES | PARMAJ | 145.37 | 18.27 | 2.35 | 3.70 |
| **2005** |  | ICP | MUSSEL | . | 9.71 | 0.53 | 2.10 | 0.34 |
| **2005** |  | ICP | MUSSEL | . | 9.61 | 0.58 | 2.11 | 0.34 |
| **2005** |  | ICP | MUSSEL | . | 9.56 | 0.59 | 2.09 | 0.34 |
| **2005** |  | ICP | MUSSEL | . | 9.58 | 0.57 | 2.10 | 0.33 |
| **2005** |  | ICP | PMWINT | . | 453.88 | 56.92 | 18.99 | 2.24 |
| **2005** |  | ICP | PMWINT | . | 488.99 | 58.76 | 17.20 | 2.26 |
| **2005** |  | ICP | PMWINT | . | 474.24 | 54.12 | 23.36 | 2.22 |
| **2005** |  | ICP | PMWINT | . | 457.47 | 44.23 | 20.80 | 2.23 |
| **2005** |  | ICP | PMWINT | . | 481.83 | 51.05 | 19.60 | 2.25 |
| **2008** | 1.02 | ICP | FECES | FICHYP | 627.03 | 82.12 | 14.45 | 5.94 |
| **2008** | 1.01 | ICP | FECES | FICHYP | 605.66 | 86.82 | 19.77 | 3.34 |
| **2008** | 1.06 | ICP | FECES | FICHYP | 341.21 | 60.59 | 7.04 | 5.74 |
| **2008** | 1.00 | ICP | FECES | FICHYP | 417.06 | 69.97 | 9.39 | 6.44 |
| **2008** | 0.91 | ICP | FECES | FICHYP | 906.10 | 200.91 | 19.75 | 6.96 |
| **2008** | 0.95 | ICP | FECES | FICHYP | 9312.81 | 307.13 | 131.31 | 4.38 |
| **2008** | 1.27 | ICP | FECES | FICHYP | 361.48 | 29.81 | 3.50 | 3.40 |
| **2008** | 1.22 | ICP | FECES | FICHYP | 317.29 | 36.43 | 5.92 | 3.19 |
| **2008** | 1.15 | ICP | FECES | FICHYP | 179.43 | 34.94 | 3.71 | 1.97 |
| **2008** | 0.90 | ICP | FECES | FICHYP | 512.51 | 116.18 | 17.93 | 4.28 |
| **2008** | 5.19 | ICP | FECES | FICHYP | 122.86 | 3.62 | 2.18 | 6.14 |
| **2008** | 5.16 | ICP | FECES | FICHYP | 110.96 | 3.99 | 1.07 | 3.01 |
| **2008** | 5.08 | ICP | FECES | FICHYP | 97.30 | 4.70 | 2.94 | 4.02 |
| **2008** | 5.04 | ICP | FECES | FICHYP | 140.26 | 6.63 | 3.14 | 3.27 |
| **2008** | 4.98 | ICP | FECES | FICHYP | 175.83 | 5.28 | 1.57 | 6.33 |
| **2008** | 11.08 | ICP | FECES | FICHYP | 180.49 | 10.44 | 2.40 | 5.30 |
| **2008** | 11.13 | ICP | FECES | FICHYP | 122.04 | 5.01 | 1.23 | 2.79 |
| **2008** | 11.11 | ICP | FECES | FICHYP | 165.24 | 7.34 | 2.10 | 2.24 |
| **2008** | 11.04 | ICP | FECES | FICHYP | 103.87 | 6.64 | 1.99 | 2.22 |
| **2008** | 10.08 | ICP | FECES | FICHYP | 91.39 | 3.69 | 1.06 | 2.44 |
| **2008** | 9.99 | ICP | FECES | FICHYP | 150.88 | 2.94 | 0.66 | 2.97 |
| **2008** | 10.13 | ICP | FECES | FICHYP | 73.42 | 1.34 | 0.53 | 2.39 |
| **2008** | 10.09 | ICP | FECES | FICHYP | 214.62 | 2.93 | 1.77 | 4.52 |
| **2008** | 10.06 | ICP | FECES | FICHYP | 167.33 | 4.42 | 1.29 | 2.18 |
| **2008** | 9.97 | ICP | FECES | FICHYP | 123.79 | 4.82 | 1.28 | 3.18 |
| **2008** | 10.00 | ICP | FECES | FICHYP | 77.61 | 4.73 | 1.70 | 1.69 |
| **2008** | 10.89 | ICP | FECES | FICHYP | 123.44 | 2.39 | 1.74 | 3.85 |
| **2008** | 11.03 | ICP | FECES | FICHYP | 96.52 | 3.40 | 0.62 | 2.48 |
| **2008** | 10.79 | ICP | FECES | FICHYP | 64.84 | 1.85 | 1.18 | 2.91 |
| **2008** | 10.68 | ICP | FECES | FICHYP | 64.82 | 2.46 | 1.68 | 5.24 |
| **2008** | 10.60 | ICP | FECES | FICHYP | 133.83 | 3.51 | 1.10 | 4.55 |
| **2008** | 5.36 | ICP | FECES | FICHYP | 151.16 | 4.19 | 3.89 | 6.04 |
| **2008** | 5.33 | ICP | FECES | FICHYP | 139.32 | 3.14 | 2.41 | 5.71 |
| **2008** | 5.36 | ICP | FECES | FICHYP | 76.14 | 3.11 | 2.09 | 1.74 |
| **2008** | 5.47 | ICP | FECES | FICHYP | 119.57 | 5.31 | 4.26 | 5.24 |
| **2008** | 5.43 | ICP | FECES | FICHYP | 104.22 | 4.19 | 13.20 | 2.59 |
| **2008** | 1.73 | ICP | FECES | FICHYP | 180.03 | 19.01 | 4.36 | 4.18 |
| **2008** | 1.81 | ICP | FECES | FICHYP | 155.54 | 44.22 | 7.73 | 2.48 |
| **2008** | 1.86 | ICP | FECES | FICHYP | 194.66 | 24.28 | 74.97 | 3.45 |
| **2008** | 1.82 | ICP | FECES | FICHYP | 148.00 | 18.16 | 7.31 | 3.98 |
| **2008** | 1.70 | ICP | FECES | FICHYP | 201.48 | 21.23 | 4.42 | 6.48 |
| **2008** | 1.61 | ICP | FECES | FICHYP | 218.90 | 40.64 | 16.80 | 4.64 |
| **2008** | 1.65 | ICP | FECES | FICHYP | 247.66 | 34.30 | 10.86 | 6.14 |
| **2008** | 1.75 | ICP | FECES | FICHYP | 196.50 | 27.27 | 3.73 | 5.12 |
| **2008** | 1.11 | ICP | FECES | FICHYP | 183.93 | 32.56 | 4.62 | 4.02 |
| **2008** | 1.12 | ICP | FECES | FICHYP | 74.87 | 18.13 | 1.97 | 1.05 |
| **2008** | 1.11 | ICP | FECES | FICHYP | 368.06 | 33.75 | 5.61 | 7.74 |
| **2008** | 1.13 | ICP | FECES | FICHYP | 213.79 | 21.65 | 6.49 | 2.60 |
| **2008** | 1.15 | ICP | FECES | FICHYP | 338.93 | 37.96 | 6.82 | 6.36 |
| **2008** | 1.33 | ICP | FECES | FICHYP | 173.05 | 18.62 | 5.21 | 4.82 |
| **2008** | 1.28 | ICP | FECES | FICHYP | 335.69 | 21.20 | 4.14 | 10.86 |
| **2008** | 1.26 | ICP | FECES | FICHYP | 229.48 | 21.39 | 3.70 | 4.91 |
| **2008** | 1.24 | ICP | FECES | FICHYP | 151.31 | 19.18 | 7.06 | 3.15 |
| **2008** | 1.23 | ICP | FECES | FICHYP | 142.94 | 11.75 | 2.58 | 2.28 |
| **2008** |  | ICP | MUSSEL | . | 10.24 | 1.32 | 2.10 | 0.35 |
| **2008** |  | ICP | MUSSEL | . | 9.87 | 1.16 | 2.08 | 0.34 |
| **2008** |  | ICP | MUSSEL | . | 9.89 | 1.05 | 2.04 | 0.36 |
| **2008** |  | ICP | MUSSEL | . | 9.95 | 1.16 | 2.08 | . |
| **2008** |  | ICP | MUSSEL | . | 9.79 | 1.15 | 1.96 | 0.33 |
| **2008** | 1.05 | ICP | FECES | PARMAJ | 93.98 | 19.63 | 1.96 | 1.24 |
| **2008** | 0.99 | ICP | FECES | PARMAJ | 146.91 | 19.37 | 9.84 | 5.51 |
| **2008** | 1.03 | ICP | FECES | PARMAJ | 173.91 | 46.36 | 13.36 | 2.00 |
| **2008** | 0.93 | ICP | FECES | PARMAJ | 210.15 | 89.97 | 8.08 | 2.01 |
| **2008** | 0.92 | ICP | FECES | PARMAJ | 153.06 | 24.86 | 21.59 | 1.32 |
| **2008** | 0.97 | ICP | FECES | PARMAJ | 247.34 | 39.94 | 16.83 | 9.62 |
| **2008** | 1.02 | ICP | FECES | PARMAJ | 255.94 | 29.35 | 12.51 | 6.18 |
| **2008** | 0.97 | ICP | FECES | PARMAJ | 310.63 | 46.53 | 17.42 | 1.52 |
| **2008** | 1.03 | ICP | FECES | PARMAJ | 132.76 | 18.52 | 3.24 | 5.85 |
| **2008** | 5.29 | ICP | FECES | PARMAJ | 112.68 | 7.45 | 3.44 | 2.39 |
| **2008** | 5.22 | ICP | FECES | PARMAJ | 105.20 | 3.19 | 0.68 | 0.26 |
| **2008** | 5.12 | ICP | FECES | PARMAJ | 121.69 | 4.36 | 1.30 | 1.78 |
| **2008** | 5.20 | ICP | FECES | PARMAJ | 123.83 | 5.80 | 4.05 | 5.67 |
| **2008** | 5.08 | ICP | FECES | PARMAJ | 114.65 | 5.89 | 2.24 | 2.23 |
| **2008** | 5.00 | ICP | FECES | PARMAJ | 61.28 | 3.33 | 1.20 | 0.63 |
| **2008** | 4.97 | ICP | FECES | PARMAJ | 73.61 | 4.07 | 1.75 | 1.37 |
| **2008** | 11.07 | ICP | FECES | PARMAJ | 96.78 | 6.10 | 2.42 | 1.97 |
| **2008** | 11.16 | ICP | FECES | PARMAJ | 64.31 | 4.85 | 2.27 | 0.65 |
| **2008** | 11.10 | ICP | FECES | PARMAJ | 56.34 | 4.36 | 1.31 | 0.49 |
| **2008** | 11.07 | ICP | FECES | PARMAJ | 43.57 | 7.94 | 3.05 | 0.56 |
| **2008** | 10.27 | ICP | FECES | PARMAJ | 45.92 | 3.34 | 1.81 | 0.52 |
| **2008** | 10.29 | ICP | FECES | PARMAJ | 32.94 | 2.49 | 3.17 | 0.58 |
| **2008** | 10.14 | ICP | FECES | PARMAJ | 103.08 | 7.81 | 1.09 | 1.68 |
| **2008** | 9.98 | ICP | FECES | PARMAJ | 80.65 | 7.14 | 1.12 | 1.02 |
| **2008** | 11.01 | ICP | FECES | PARMAJ | 47.34 | 2.48 | 0.97 | 0.76 |
| **2008** | 10.97 | ICP | FECES | PARMAJ | 53.58 | 19.28 | 0.96 | 0.41 |
| **2008** | 10.76 | ICP | FECES | PARMAJ | 54.64 | 3.89 | 1.41 | 1.93 |
| **2008** | 10.67 | ICP | FECES | PARMAJ | 68.62 | 3.73 | 1.84 | 1.37 |
| **2008** | 10.55 | ICP | FECES | PARMAJ | 54.18 | 5.92 | 1.71 | 0.89 |
| **2008** | 10.58 | ICP | FECES | PARMAJ | 64.12 | 4.53 | 2.39 | 1.15 |
| **2008** | 5.60 | ICP | FECES | PARMAJ | 81.05 | 4.54 | 1.51 | 1.12 |
| **2008** | 5.49 | ICP | FECES | PARMAJ | 34.43 | 3.40 | 0.75 | 0.34 |
| **2008** | 5.36 | ICP | FECES | PARMAJ | 68.25 | 2.52 | 0.70 | 1.31 |
| **2008** | 5.48 | ICP | FECES | PARMAJ | 57.20 | 3.27 | 0.73 | 2.16 |
| **2008** | 1.81 | ICP | FECES | PARMAJ | 69.68 | 11.60 | 3.70 | 1.27 |
| **2008** | 1.89 | ICP | FECES | PARMAJ | 134.09 | 6.43 | 2.03 | 1.04 |
| **2008** | 1.96 | ICP | FECES | PARMAJ | 103.07 | 5.21 | 1.52 | 1.01 |
| **2008** | 1.71 | ICP | FECES | PARMAJ | 272.18 | 55.04 | 6.36 | 2.74 |
| **2008** | 1.61 | ICP | FECES | PARMAJ | 169.49 | 35.94 | 6.02 | 1.75 |
| **2008** | 1.61 | ICP | FECES | PARMAJ | 263.35 | 45.60 | 5.89 | 2.66 |
| **2008** | 1.62 | ICP | FECES | PARMAJ | 237.41 | 45.04 | 7.53 | 3.29 |
| **2008** | 1.63 | ICP | FECES | PARMAJ | 172.25 | 40.69 | 3.73 | 2.77 |
| **2008** | 1.64 | ICP | FECES | PARMAJ | 164.48 | 15.96 | 3.23 | 4.94 |
| **2008** | 1.74 | ICP | FECES | PARMAJ | 184.05 | 23.64 | 6.43 | 9.95 |
| **2008** | 1.57 | ICP | FECES | PARMAJ | 143.78 | 26.04 | 14.20 | 2.49 |
| **2008** | 1.15 | ICP | FECES | PARMAJ | 97.67 | 13.09 | 1.76 | 3.23 |
| **2008** | 1.38 | ICP | FECES | PARMAJ | 181.57 | 27.16 | 4.50 | 2.63 |
| **2008** | 1.30 | ICP | FECES | PARMAJ | 148.36 | 13.56 | 1.90 | 2.48 |
